# Vitamin D Potentiates the Inhibitory Effect of MicroRNA-130a in Hepatitis C Virus Replication Independent of Type I Interferon Signaling Pathway

**DOI:** 10.1155/2015/508989

**Published:** 2015-04-28

**Authors:** Xiaoqiong Duan, Yujuan Guan, Yujia Li, Shan Chen, Shilin Li, Limin Chen

**Affiliations:** ^1^Institute of Blood Transfusion, Chinese Academy of Medical Sciences and Peking Union Medical College, Chengdu, China; ^2^Guangzhou No. 8 People's Hospital, Guangzhou, China; ^3^Toronto General Research Institute, University of Toronto, Toronto, ON, Canada

## Abstract

Calcitriol, the bioactive metabolite of vitamin D, was reported to inhibit HCV production in a synergistic fashion with interferon, a treatment *in vitro*. Our previous study established that miR-130a inhibits HCV replication by restoring the host innate immune response. We aimed to determine whether there is additive inhibitory effect of calcitriol and miR-130a on HCV replication. Here we showed that calcitriol potentiates the anti-HCV effect of miR-130a in both Con1b replicon and J6/JFH1 culture systems. Intriguingly, this potentiating effect of calcitriol on miR-130a was not through upregulating the expression of cellular miR-130a or through increasing the miR-130a-mediated IFN*α*/*β* production. All these findings may contribute to the development of novel anti-HCV therapeutic strategies although the antiviral mechanism needs to be further investigated.

## 1. Introduction 

Hepatitis C Virus (HCV) infection and associated liver diseases are a global health problem. According to WHO's latest report, approximately 130–150 million people are chronically infected with HCV worldwide and 350 000 to 500 000 people died each year from HCV-related liver diseases (http://www.who.int). Pegylated interferon-alpha (IFN*α*)/ribavirin therapy is still the mainstay in the management of HCV infection in most developing countries, though several direct acting antiviral drugs (DAAs) have been approved by FDA since 2011 [1].

Vitamin D plays a central role in calcium and phosphate homeostasis [2]. There are two forms of vitamin D, vitamin D_2_ and vitamin D_3_, with the latter being dominant in mammals [3]. Calcitriol, 1*α*, 25-Dihydroxyvitamin D3, the bioactive metabolite of vitamin D, is generated through two successive hydroxylations [4]. Firstly, vitamin D derived from the action of sunlight on the epidermis or obtained from diet is hydroxylated in the liver to form 25-hydroxyvitamin D, the main circulating form of vitamin D. Then 25-hydroxyvitamin D undergoes a second hydroxylation to form calcitriol in kidney [4, 5]. Recently, vitamin D and its metabolites were found to play important roles in the host anti-HCV response [4, 6–8]. Vitamin D supplementation has been shown to improve the efficacy of anti-HCV therapy with IFN and ribavirin [7, 8]. Further investigation demonstrated that vitamin D and its metabolites significantly inhibit HCV production* in vitro* [4, 6] and it acts with interferon synergistically [8].

MicroRNA is a class of small (~22 nt) noncoding RNAs that serve as posttranscriptional regulators of gene expression [9]. In recent years, a number of miRNAs associated with HCV infection have been reported [10–14]. Our previous study established that miR-130a inhibits HCV replication by restoring the host innate immune response [12]. Overexpression of miR-130a inhibited HCV RNA replication and stimulated the expression of IFN*α* and IFN*β* both in Con1b replicon cells and in J6/JFH1 HCVcc system [12]. However, the underlying mechanisms are incompletely understood.

In this current study, we aim to explore the potential inhibitory effect of calcitriol, the bioactive vitamin D metabolite, alone or in combination with miR-130a on HCV RNA replication* in vitro*. Our data indicated that treatment with calcitriol or miR-130a alone inhibited HCV replication and combination of these two showed additive inhibitory effect. Further studies revealed that the additive effect of calcitriol on miR-130a was not through upregulating the expression of cellular miR-130a or increasing the miR-130a-mediated IFN*α*/*β* production. More detailed studies are needed to understand the mechanisms of this additive effect in suppressing HCV production.

## 2. Materials and Methods

### 2.1. Reagents

Calcitriol (1*α*,25-Dihydroxyvitamin D_3_, D1530) was purchased from Sigma Chemical (St. Louis, MO). It was dissolved in ethanol at the stock concentration of 500 *μ*M stored at −20°C in dark until use. miR-130a (miRIDIAN mimic hsa-miR-130a; C-300598-03-0005) and negative control miRNA (miRIDIAN mimic negative control; CN-001000-01) were purchased from Dharmacon (Lafayette, CO).

### 2.2. Virus and Cell Lines

The Huh7.5.1 cell line was kindly provided by Professor Zhongtian Qi (the Second Military Medical University, Shanghai, China). Con1b subgenomic genotype 1b HCV replicon cell line was obtained from Dr. Ian McGilvray (University of Toronto, Canada). The Con1b cell line is a Huh7.5 cell population containing the full-length HCV genotype 1b replicon in which HCV RNA replicates although no infectious virus particles were produced. HCV infectious clone J6/JFH1, the full-length chimerical genome from the infectious JFH1 (genotype 2a), was generously provided by Dr. Charles Rice (Rockefeller University). Cell culture and HCV replication assays were carried out as described in our previous publication [12].

### 2.3. miR-130a Transfection and Calcitriol Treatment

Huh7.5.1 cells were seeded and infected with HCV J6/JFH1 stock as described [12]. The cells were treated with various concentrations of calcitriol (0, 0.05, 0.1, 0.15, 0.2, and 0.4 *μ*M) or transfected with miR-130a 4 h after infection. Transfection with miR-130a mimic or miRNA mimic negative control (Nc) (final concentration 2 nM) was performed using DharmaFECT 4 (Dharmacon, Lafayette, CO, USA) according to the manufacturer's instruction. For combination treatment of calcitriol with miR-130a, calcitriol was added after miR-130a transfection with a final concentration of 0.1 *μ*M. Cells were harvested 48 h later, and total RNA was extracted as described below.

### 2.4. RNA Isolation, Reverse Transcription, and Real-Time PCR

Total RNA was isolated using TRIzol method (Invitrogen, Carlsbad, CA, USA) according to the manufacturer's instructions. The first-strand complementary DNA (cDNA) was synthesized for gene expression analysis and miRNA expression using random primer (Roche, Basel, Switzerland) and miR-130a-specific reverse transcription primer (RiboBio Co., Ltd., Guangzhou, China), respectively. The real-time RT-PCR for the quantification of HCV, IFN*α*, IFN*β*, and GAPDH mRNAs was performed with the FastStart Universal SYBR Green Master Mix (Roche). All the assays were performed as described [12].

### 2.5. Statistical Analyses

Data were statistically analysed by an ANOVA with post hoc analysis, and *P* values less than 0.05 were considered statistically significant. All data are representative of at least three repeated experiments.

## 3. Results

### 3.1. Calcitriol Inhibits HCV RNA Replication

**Figure 1:**
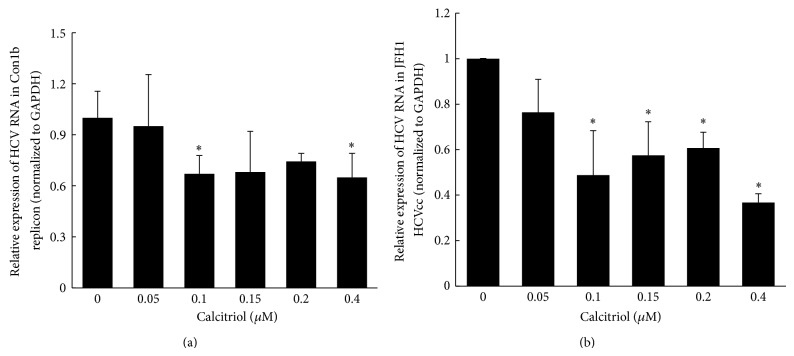
Calcitriol inhibits HCV RNA replication both in Con1b replicon and in J6/JFH1 HCVcc. Huh7.5-Con1b replicon cells (a) or J6/JFH1 infected HCVcc (b) were treated with 0–0.4 *μ*M calcitriol for 48 h, after which cells were harvested and total RNA was extracted. The levels of HCV RNA and GAPDH mRNA were measured by quantitative RT-PCR as described in Section 2. Data are presented as means ± SD, *n* = 3. ^∗^ *P* < 0.05 versus 0 *μ*M (untreated control).

Huh7.5 cells harboring subgenomic HCV replicons (HCV Con1b) or Huh7.5.1 cells infected with HCVcc were exposed to increasing concentrations (0–0.4 *μ*M) of calcitriol for 48 h and HCV RNAs were quantified by quantitative RT-PCR. Firstly, we excluded the influence of ethanol on HCV replication at the same concentration used in dissolving calcitriol in this study (see supplementary Figure 1 in Supplementary Material available online at http://dx.doi.org/10.1155/2015/508989). As shown in Figure 1, calcitriol inhibited HCV RNA replication both in Con1b replicon and in HCVcc systems. The inhibitory effect was increased as the concentration of calcitriol was below 0.1 *μ*M and then fluctuated with the increase of calcitriol concentration. 0.1 *μ*M of calcitriol resulted in a significant inhibition in HCV RNA replication compared with the control (*P* < 0.05). HCV RNA replication was inhibited ~33% in Con1b replicon and ~52% in HCVcc, respectively. As such 0.1 *μ*M of calcitriol was used in the following assays.

### 3.2. Calcitriol Enhanced the Antiviral Effect of miR-130a

**Figure 2:**
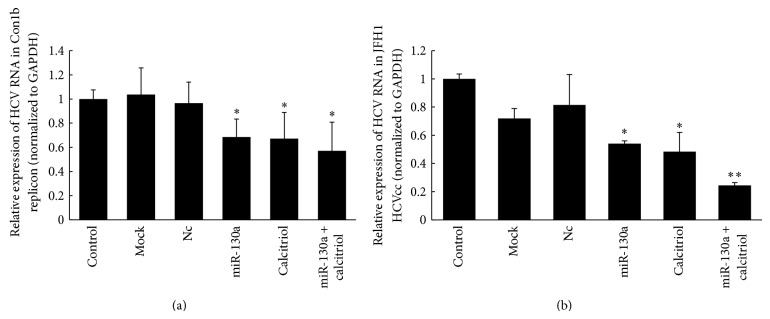
Calcitriol potentiates anti-HCV effect of miR-130a in both Con1b replicon (a) and J6/JFH1 culture systems (b). Huh7.5-Con1b replicon cells (a) or J6/JFH1 infected HCVcc (b) were transfected with 2 nM miR-130a mimic or treated with 0.1 *μ*M calcitriol or both and HCV RNAs were quantified by quantitative RT-PCR 48 h after transfection as described in Section 2. Data are presented as means ± SD, *n* = 3. ^∗^ *P* < 0.05; ^∗∗^ *P* < 0.01 versus control and mock and Nc (microRNA mimic negative control).

In our previous study, we found overexpression of miR-130a upregulated the expression of IFN*α* and IFN*β* [12]. Interestingly, it was reported that calcitriol inhibited HCV production through enhancing IFN signaling in HCV-infected cells [4, 15]. To explore whether calcitriol has synergistic effect with miR-130a in inhibiting HCV replication, we treated Con1b HCV replicon cells and HCV-infected cells with a combination of both agents. Cells were transfected with 2 nM miR-130a mimic or treated with 0.1 *μ*M calcitriol or both; total RNAs were extracted by TRIzol and HCV RNAs were quantified by quantitative RT-PCR 48 h after transfection. As shown in Figure 2, combination treatment of miR-130a and calcitriol resulted in an additive effect on HCV RNA replication in J6/JFH1 HCV culture system and a subadditive effect in Con1b replicon cells. The inhibitory effect reached 43% in Con1b replicon cells after combination treatment compared with miR-130a alone (~32%) and calcitriol alone (~33%). In HCVcc, the additive effect was even more robust, resulting in ~76% inhibition after combination treatment compared with miR-130a alone (~46%) or calcitriol alone (~52%).

### 3.3. Both Calcitriol and miR-130a Stimulate Type I IFN Production

**Figure 3:**
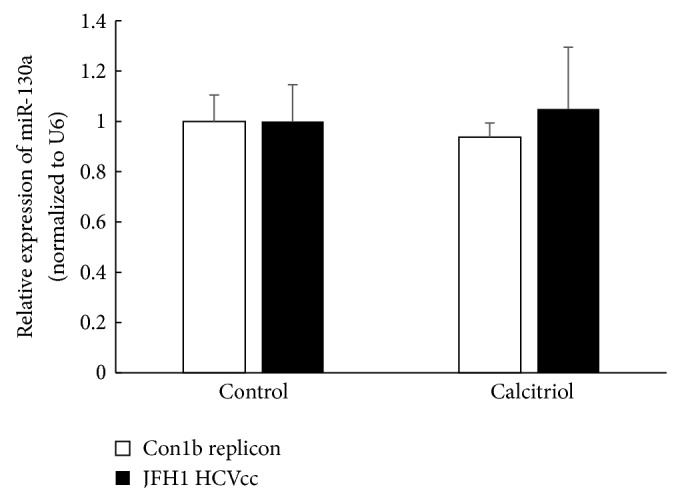
Calcitriol has no effect on the expression of miR-130a. Huh7.5-Con1b replicon cells (a) or J6/JFH1 infected HCVcc (b) were treated with 0.1 *μ*M calcitriol for 48 h; the expressions of miR-130a were quantified by quantitative RT-PCR as described in Section 2. Data are presented as means ± SD, *n* = 3.

**Figure 4:**
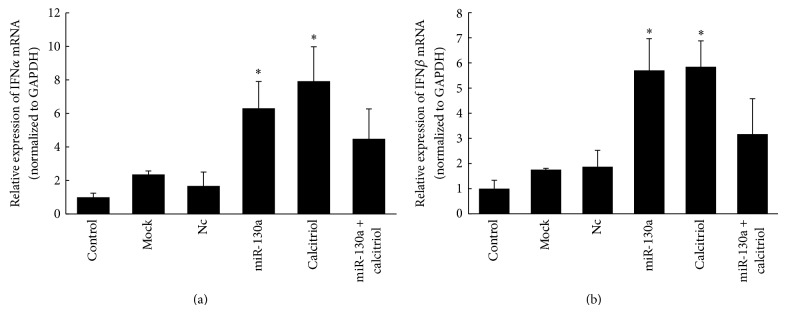
Calcitriol does not increase the miR-130a-induced IFN*α* and IFN*β* upregulation. Huh7.5.1 cells infected with J6/JFH1 infected HCVcc were transfected with 2 nM miR-130a mimic or treated with 0.1 *μ*M calcitriol or both and the expressions of IFN*α* (a) and IFN*β* (b) mRNAs were quantified by quantitative RT-PCR 48 h after transfection as described in Section 2. Data are presented as means ± SD, *n* = 3. ^∗^ *P* < 0.05 versus control and mock and Nc (microRNA mimic negative control).

To explore the mechanism of the enhanced antiviral effect of miR-130a in the presence of calcitriol, we first determined the expression level of miR-130a in Con1b replicon cells and naive Huh7.5.1 cells in the presence or absence of calcitriol. As shown in Figure 3, miR-130a expression was not affected by calcitriol treatment. Ethanol has no effect on the expression of IFN*α*/*β* at the same concentration used in dissolving calcitriol in this study (see supplementary Figure 1). We then tested whether combination of miR-130a with calcitriol may enhance IFN-mediated suppression of HCV replication. As shown in Figure 4, both calcitriol and miR-130a upregulated type I IFN production significantly. The expression of IFN*α* increased by ~6.3-fold after being transfected with miR-130a mimic and ~7.9-fold after being treated with calcitriol. A similar upregulated expression of IFN*β* was also observed, with ~5.7-fold after miR-130a overexpression and ~5.8-fold after calcitriol treatment. Notably, instead of an additive effect in upregulation of type I IFN production, adding the two together abrogated pro-IFN effect of either calcitriol or miR-130a.

## 4. Discussion

Recently, vitamin D and its metabolites were reported to play important roles in immunomodulation [16]. It was reported that low serum vitamin D levels are associated with severe fibrosis and poor responsiveness to IFN-based therapy in genotype 1 chronic hepatitis C patients [17]. Calcitriol is the bioactive metabolite of vitamin D. However, there are some controversies about whether calcitriol has a direct anti-HCV effect. In this current study, we demonstrated that calcitriol significantly inhibits HCV RNA replication in both Con1b replicon cells and J6/JFH1 HCVcc. Our results are in line with a previous study by Gal-Tanamy et al. [4].

Other studies also established that calcitriol enhanced the anti-HCV effect of IFN*α* [4, 15], and our results showed the same upregulated expression of IFN*α*/*β*. In our previous published study, we also observed that miR-130a suppressed HCV replication and stimulated IFN*α*/*β* expression significantly [12]. All these findings led us to surmise that calcitriol may have an additive effect with miR-130a. As expected, the combination of miR-130a and calcitriol resulted in a more pronounced suppression of HCV replication, especially in HCVcc system.

Next, we investigated whether this additive effect was caused by the additive effect of the two agents on the IFN pathway because either calcitriol or miR-130a stimulated IFN*α*/*β* production. Surprisingly, the expression of IFN*α*/*β* did not increase more following combination treatment of miR-130a and calcitriol but decreased comparing with either miR-130a or calcitriol. This result indicated that adding the two together abrogated the pro-IFN effect of either calcitriol or miR-130a, implying that the inhibitory effect of calcitriol or miR-130a on HCV may function through other mechanisms in addition to the activation of the IFN-dependent pathways. They may inhibit virus entry or assembly as the two together inhibit HCVcc more than replicon system as shown in Figure 2. In addition, it was found that vitamin D plays an important role in human T cells activation [18], indicating the potential role of vitamin D in anti-HCV therapy deserved further investigation.

## Supplementary Material

Ethanol has no effect on the expressions of HCV RNA and IFNα/β at the concentration of 0.02%.
As the calcitriol was dissolved in 100% ethanol at the stock concentration of 500 ωM and used at the concentration of 0.1ωM, the final concentration of ethanol in our experiment was 0.02%. In order to make sure there is no effect of 0.02% ethanol on HCV replication and IFNα/β expression, Con1b replicon cells and Huh7.5.1 cells infected with J6/JFH1 HCVcc were seeded in a 24-well plate for overnight and 5 ul of 2% ethanol was added into each well (final concentration 0.02%). Total RNAs were extracted 48h post treatment and expression levels of HCV RNA, IFNα and IFNβ mRNAs were quantified by RT-PCR. The results showed that ethanol has no effect on the expressions of HCV RNA and IFNα/β at the concentration of 0.02%.

## References

[1] Chandra P. K., Gunduz F., Hazari S. (2014). Impaired expression of type I and type II interferon receptors in HCV-associated chronic liver disease and liver cirrhosis. *PLoS ONE*.

[2] Brown A. J., Dusso A., Slatopolsky E. (1999). Vitamin D. *The American Journal of Physiology—Renal Physiology*.

[3] Gilliland D. L., Black C. K., Denison J. E., Seipelt C. T., Baugh S. (2013). Simultaneous determination of vitamins D2 and D3 by electrospray ionization LC/MS/MS in infant formula and adult nutritionals: first action 2012.11. *Journal of AOAC International*.

[4] Gal-Tanamy M., Bachmetov L., Ravid A. (2011). Vitamin D: an innate antiviral agent suppressing hepatitis C virus in human hepatocytes. *Hepatology*.

[5] Hewison M. (2012). An update on vitamin D and human immunity. *Clinical Endocrinology*.

[6] Matsumura T., Kato T., Sugiyama N. (2012). 25-hydroxyvitamin D3 suppresses hepatitis C virus production. *Hepatology*.

[7] Bitetto D., Fabris C., Fornasiere E. (2011). Vitamin D supplementation improves response to antiviral treatment for recurrent hepatitis C. *Transplant International*.

[8] Abu-Mouch S., Fireman Z., Jarchovsky J., Zeina A.-R., Assy N. (2011). Vitamin D supplementation improves sustained virologic response in chronic hepatitis C (genotype 1)-naïve patients. *World Journal of Gastroenterology*.

[9] Bartel D. P. (2004). MicroRNAs: genomics, biogenesis, mechanism, and function. *Cell*.

[10] Jopling C. L. (2010). Targeting microRNA-122 to treat hepatitis C virus infection. *Viruses*.

[11] Bala S., Tilahun Y., Taha O. (2012). Increased microRNA-155 expression in the serum and peripheral monocytes in chronic HCV infection. *Journal of Translational Medicine*.

[12] Li S., Duan X., Li Y., Liu B., McGilvray I., Chen L. (2014). MicroRNA-130a inhibits HCV replication by restoring the innate immune response. *Journal of Viral Hepatitis*.

[13] Chen Y., Chen J., Wang H. (2013). HCV-induced miR-21 contributes to evasion of host immune system by targeting MyD88 and IRAK1. *PLoS Pathogens*.

[14] Xu G., Yang F., Ding C. L. (2014). MiR-221 accentuates IFNs anti-HCV effect by downregulating SOCS1 and SOCS3. *Virology*.

[15] Lange C. M., Gouttenoire J., Duong F. H., Morikawa K., Heim M. H., Moradpour D. (2014). Vitamin D receptor and Jak-STAT signaling crosstalk results in calcitriol-mediated increase of hepatocellular response to IFN-*α*. *The Journal of Immunology*.

[16] Nagpal S., Na S., Rathnachalam R. (2005). Noncalcemic actions of vitamin D receptor ligands. *Endocrine Reviews*.

[17] Petta S., Cammà C., Scazzone C. (2010). Low vitamin d serum level is related to severe fibrosis and low responsiveness to interferon-based therapy in genotype 1 chronic hepatitis C. *Hepatology*.

[18] Von Essen M. R., Kongsbak M., Schjerling P., Olgaard K., Ødum N., Geisler C. (2010). Vitamin D controls T cell antigen receptor signaling and activation of human T cells. *Nature Immunology*.

