# Supplementary figures and images for: Vitamin D Potentiates the Inhibitory Effect of MicroRNA-130a in Hepatitis C Virus Replication Independent of Type I Interferon Signaling Pathway
Source: Mediators Inflamm. 2015 Apr 28;2015:508989. doi: 10.1155/2015/508989 (PMC4427768; doi:10.1155/2015/508989)

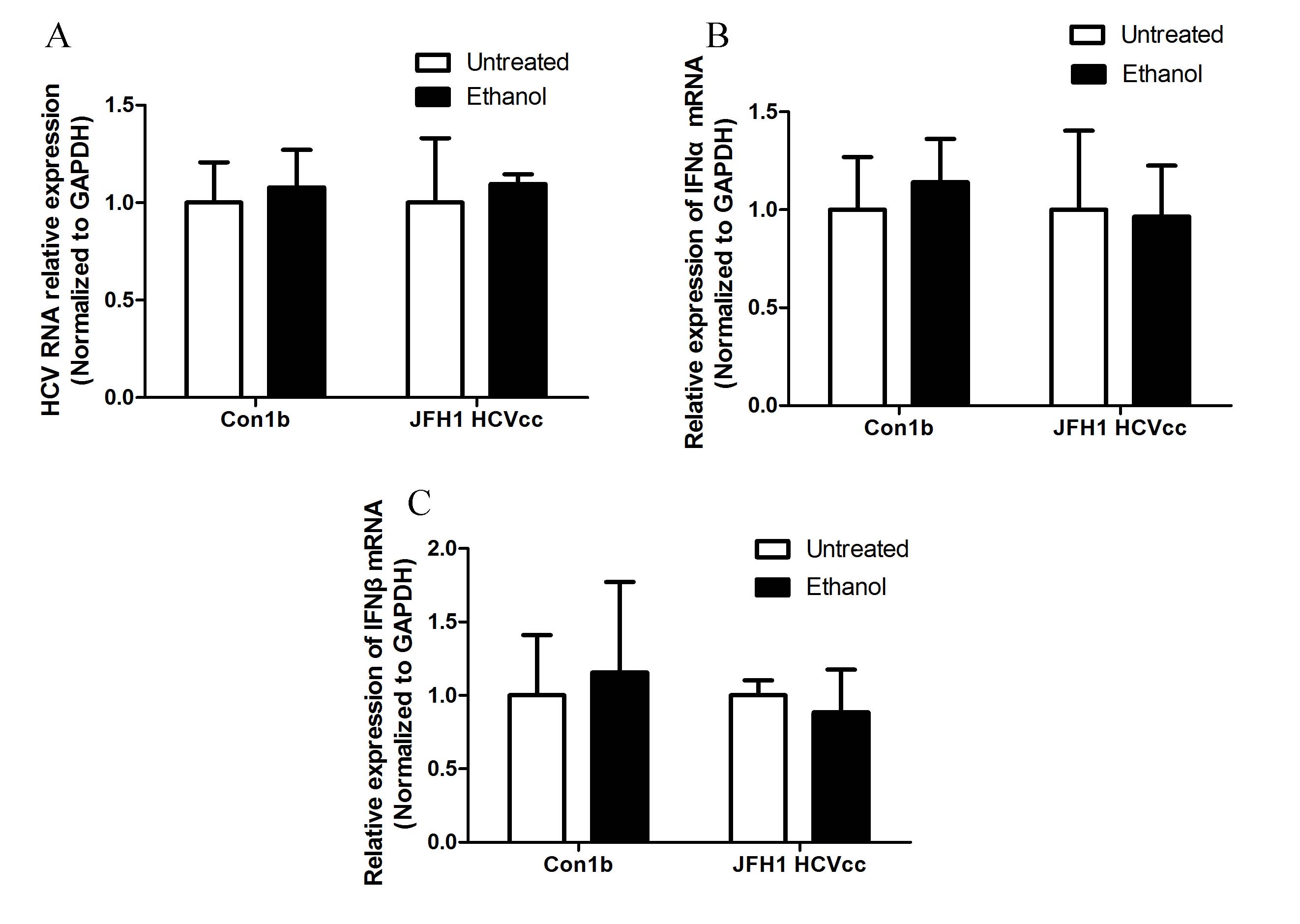

Supplement: Supplementary file 1 — Ethanol has no effect on the expressions of HCV RNA and IFNα/β at the concentration of 0.02%. As the calcitriol was dissolved in 100% ethanol at the stock concentration of 500 ωM and used at the concentration of 0.1ωM, the final concentration of ethanol in our experiment was 0.02%. In order to make sure there is no effect of 0.02% ethanol on HCV replication and IFNα/β expression, Con1b replicon cells and Huh7.5.1 cells infected with J6/JFH1 HCVcc were seeded in a 24-well plate for overnight and 5 ul of 2% ethanol was added into each well (final concentration 0.02%). Total RNAs were extracted 48h post treatment and expression levels of HCV RNA, IFNα and IFNβ mRNAs were quantified by RT-PCR. The results showed that ethanol has no effect on the expressions of HCV RNA and IFNα/β at the concentration of 0.02%. [file 508989.f1.jpg]
